# Assessment of ventilation in low-resource healthcare settings: Montserrado County, Liberia—2022−2023

**DOI:** 10.1017/ash.2023.225

**Published:** 2023-09-29

**Authors:** Krithika Srinivasan, Ronan Arthur, Ashley Styczynski, Ethan Bell, Thomas Baer, Jorge Salinas

## Abstract

**Background:** Mitigating the risk of nosocomial respiratory disease transmission in the healthcare facilities of low- and middle−income countries (LMICs) poses unique challenges because mechanical ventilation and mixed−mode strategies are often unavailable. Carbon dioxide (CO_2_) can serve as a proxy for ventilation and, hence, airborne infectious disease transmission risk in naturally ventilated spaces. We assessed the adequacy of ventilation in Liberian hospitals. **Methods:** We sampled 3 hospitals, both urban and rural, in Montserrado County, Liberia. Moreover, 3 CO_2_ meters were concurrently utilized to measure CO_2_ levels at a 1-meter height in every patient-care room in each facility. We recorded temperature, humidity, room dimensions, and number of people in the rooms. From these variables, we calculated absolute ventilation using the ASHRAE equation to determine areas with the highest risk of nosocomial respiratory disease transmission. We also recorded qualitative observations about the sampled spaces. **Results:** From August 2022 to February 2023, 39 rooms in 3 healthcare facilities were sampled. Initial quantitative findings show that only 8 rooms (21%) met the WHO-recommended ventilation rate of 60 L per second per person. The average ventilation rate per person in the adequately ventilated settings was 86 L per second per patient, compared to 19 liters per second per patient in inadequately ventilated rooms. Additionally, 467 ppm mean CO_2_ was noted in well-ventilated rooms compared to 895 ppm mean CO_2_ in inadequately ventilated rooms.

Initial qualitative observations showed that facilities with lower CO_2_ readings tended to be older constructions that likely had been constructed with airborne disease such as tuberculosis in mind. Willingness to open windows was limited by lack of window screens for malaria prevention, and there was a pervasive fallacy that air conditioning was a source of ventilation. Correspondingly, of the 31 inadequately ventilated rooms, 22 (71%) had operating air conditioning units compared with 4 (50%) of the 8 adequately ventilated rooms. Overall, of the 13 rooms without air conditioning, 7 (54%) were more frequently characterized by open windows compared to only 5 of 26 (28%) of rooms that did have air conditioners. **Conclusions:** Being prepared for the next respiratory disease outbreak and creating more resilient healthcare systems in LMICs requires a frameshift of prevention strategies. Measuring CO_2_ provides a simple strategy for identifying areas at highest risk for nosocomial respiratory disease transmission, which can be prioritized for low-cost environmental interventions, such as provision of window screens, as part of routine infection prevention and control efforts.

**Figure:**
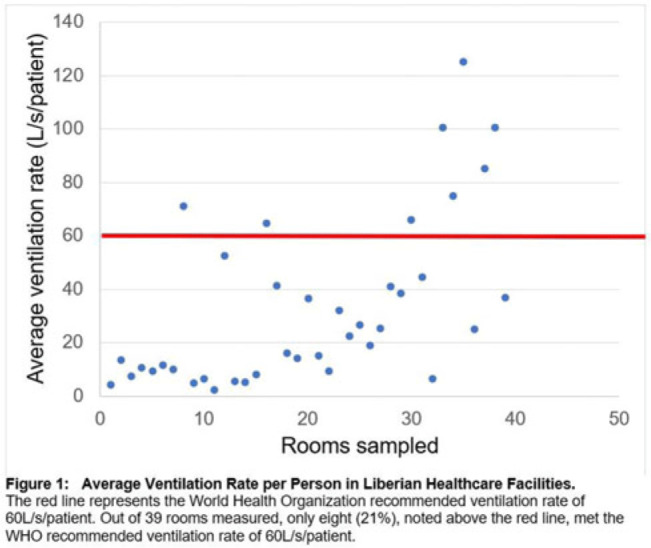

**Disclosure:** None

